# Impaired Priming of SARS-CoV-2-Specific Naive CD8^+^ T Cells in Older Subjects

**DOI:** 10.3389/fimmu.2021.693054

**Published:** 2021-07-13

**Authors:** Eleonora Gallerani, Davide Proietto, Beatrice Dallan, Marco Campagnaro, Salvatore Pacifico, Valentina Albanese, Erika Marzola, Peggy Marconi, Antonella Caputo, Victor Appay, Riccardo Gavioli, Francesco Nicoli

**Affiliations:** ^1^ Laboratory of Biochemistry, Immunology and Microbiology (BIM), Department of Chemical, Pharmaceutical and Agricultural Sciences, University of Ferrara, Ferrara, Italy; ^2^ Department of Chemical, Pharmaceutical and Agricultural Sciences, University of Ferrara, Ferrara, Italy; ^3^ CNRS UMR 5164, ImmunoConcEpT, Université de Bordeaux, Bordeaux, France

**Keywords:** SARS-CoV-2, immune aging, naive T cells, cellular immunity, epitopes, primary responses, CD8^+^ T cells

## Abstract

Advanced age is associated with severe symptoms and death upon SARS-CoV-2 infection. Virus-specific CD8^+^ T-cell responses have shown to be protective toward critical COVID-19 manifestations, suggesting that suboptimal cellular immunity may contribute to the age-pattern of the disease. The induction of a CD8^+^ T-cell response against an emerging pathogen like SARS-CoV-2 relies on the activation of naive T cells. To investigate whether the primary CD8^+^ T-cell response against this virus is defective in advanced age, we used an *in vitro* approach to prime SARS-CoV-2-specific naive CD8^+^ T cells from healthy, unexposed donors of different age groups. Compared to younger adults, older individuals display a poor SARS-CoV-2-specific T-cell priming capacity in terms of both magnitude and quality of the response. In addition, older subjects recognize a lower number of epitopes. Our results implicate that immune aging is associated with altered primary SARS-CoV-2-specific CD8^+^ T-cell responses.

## Introduction

SARS-CoV-2 is the causative agent of the coronavirus disease 2019 (COVID-19), whose mortality rate progressively increases with age (1). Although clear correlates of protection are still unknown, studies suggest that an uncoordinated adaptive immunity, and in particular the lack of virus-specific cellular responses, is associated with severe forms of disease (2, 3). Lower frequencies of SARS-CoV-2 specific CD8^+^ T cells have been found in critically ill patients (4, 5), while the onset of wide cellular responses precedes infection resolution (6). Furthermore, SARS-CoV-2-specific memory CD8^+^ T cells seem important for protection against reinfections (7).

The functional capacity of SARS-CoV-2-specific T cells is lower in elderly patients (2, 8), which harbor poor virus-specific CD8^+^ T-cell responses (4). As a new threat emerged in 2019, the induction of SARS-CoV-2-specific cellular immunity relies mainly on the activation of naive T cells. This subset progressively decreases with age (9–11), exposing elderly people to higher risks of severe consequences upon infection with emerging pathogens (12).

To evaluate whether the age impacts on primary SARS-CoV-2-specific responses, we exploited an *in vitro* T-cell priming approach to stimulate naive precursors from unexposed subjects of different age groups. Owing to this disease-free system, which permits focusing solely on immune cell features without other confounders (*e.g.* physiological stressors induced by the disease), we could observe that the induction of *de novo* SARS-CoV-2-specific T-cell immunity is altered with aging, resulting in narrower and less functional responses.

## Materials and Methods

### Subjects and Peripheral Blood Mononuclear Cell Isolation

Peripheral blood samples (n = 19) were obtained in an anonymous fashion from blood donors through the Blood Bank of the Ferrara Hospital. The protocol was approved by the Regional Health Authority (AUSL), which supervises blood donations. PBMCs were isolated by Ficoll-Paque (GE-Healthcare, Milan, Italy) density gradient centrifugation as previously described (13). Donors were either sampled before December 2019 or during 2020. In the latter case, donors were serologically negative for SARS-CoV-2 antibodies. PBMCs were stored in liquid nitrogen.

### Peptides

The following peptides were synthesized by solid phase method and purified by High Performance Liquid Chromatography (HPLC) to >97% purity: 37 SARS-CoV-2-derived peptides, the EV10 peptide (ELAGIGILTV) and the peptides used to stimulate memory responses (NLVPMVATV from (cytomegalovirus) CMV; CLGGLLTMV and GLCTLVAML from (Epstein-Barr Virus) EBV; ALMLRLLRI, NLLTTPKFT, RMLGDVMAV, FLGAGALAV, ALLGLTLGV and GIFEDRAPV from (Herpes simplex virus type 1) HSV-1). The SARS-CoV-2-derived peptides were selected from wider lists of predicted and/or confirmed epitopes, as indicated in **Table 1** and **Supplementary Table S1**. Peptides, either as single or in pool/matrixes, were suspended in DMSO and used at the concentration of 1 μM. Matrixes were composed of six peptides each (**Supplementary Table S1**).

**Table 1:** Responses to the HLA-A2-restricted, SARS-CoV-2-derived peptides used and homology with HCoVs.

Antigen		Responders			Literature	% of homology with HCoV			
Prot.	Code	Tot, n=19	Mid, n=10	Old, n = 9	Reported Frequency^a^	NL63	229E	OC43	HKU1
**E**	**SLV**	**8**	7	1	Not tested	22.2	22.2	44.4	44.4
**M**	**KLL**	**4**	4	0	N (14)	33.3	33.3	44.4	33.3
					L (15)				
					L (16)				
**M**	**TLA**	**6**	6	0	N (17)	20	20	20	10
					N (18)				
**M**	**GLM**	**5**	3	2	N (16)	33.3	44.4	44.4	33.3
					L (19)				
**N**	**ALN**	**5**	4	1	N (20)	11.1	22.2	66.6	44.4
					N (17)				
**N**	**LQL**	**2**	1	1	N (20)	22.2	44.4	44.4	44.4
					N (17)				
					N (14)				
**N**	**LAL**	**4**	1	3	N (20)	22.2	22.2	11.1	11.1
					L (17)				
					H (21)				
**N**	**LLL**	**5**	5	0	N (20)	22.2	22.2	11.1	11.1
					N (18)				
					N (21)				
					L (17)				
					L (4)				
					L (6)				
					L (16)				
					H (14)				
**N**	**RLN**	**3**	3	0	Not tested	0	11.1	11.1	11.1
**N**	**GMS**	**4**	3	1	L (33)	11.1	11.1	22.2	11.1
					L (17)				
					L (15)				
					L (16)				
**N**	**ILL**	**4**	3	1	L (20)	22.2	11.1	33.3	22.2
**RdRp**	**NLI**	**3**	2	1	N (6)	44.4	22.2	44.4	44.4
**RdRp**	**YTM**	**4**	3	1	L (15)	80	70	80	80
**RdRp**	**SLL**	**2**	2	0	Not tested	55.5	33.3	55.5	55.5
**RdRp**	**KIF**	**3**	3	0	Not tested	70	70	90	90
**RdRp**	**RLA**	**2**	0	2	Not tested	77.7	77.7	100	100
**RdRp**	**YLP**	**4**	4	0	Not tested	100	81.8	81.8	100
**RdRp**	**LMI**	**3**	2	1	N (17)	55.5	55.5	88.8	88.8
**RdRp**	**MLD**	**7**	6	1	Not tested	44.4	44.4	66.6	66.6
**S**	**TLD**	**6**	4	2	N (14)	11.1	0	11.1	0
					L (16)				
**S**	**YLQ**	**5**	4	1	L (16)	0	0	44.4	44.4
					H (22)				
					H (4)				
					H (15)				
					H (6)				
					H (18)				
**S**	**KIA**	**7**	5	2	N (42)	22.2	11.1	22.2	33.3
					L (22)				
**S**	**KLP**	**6**	4	2	N (16)	22.2	33.3	22.2	22.2
					L (22)				
**S**	**SII**	**5**	2	3	N (22)	0	0	11.1	22.2
					L (15)				
					L (19)				
					L (16)				
**S**	**LLF**	**6**	5	1	N (16)	55.5	33.3	66.6	66.6
					L (22)				
**S**	**ALN**	**4**	2	2	N (20)	66.6	55.5	66.6	66.6
					N (17)				
					N (18)				
					L (22)				
**S**	**VLN**	**4**	3	1	N (17)	33.3	33.3	66.6	66.6
					N (16)				
					L (22)				
					H (15)				
					H (19)				
**S**	**RLD**	**4**	3	1	N (16)	44.4	44.4	66.6	66.6
**S**	**RLQ**	**2**	2	0	N (18)	44.4	44.4	55.5	55.5
					L (16)				
					H (22)				
**S**	**HLM**	**4**	4	0	L (16)	44.4	33.3	44.4	44.4
**S**	**VVF**	**1**	1	0	N (6),	33.3	44.4	44.4	33.3
					L (16)				
**S**	**RLN**	**5**	4	1	N (17)	11.1	22.2	55.5	33.3
					N (16)				
					N (18)				
					L (22)				
**S**	**NLN**	**4**	3	1	N (22)	33.3	55.5	55.5	55.5
					N (17)				
					N (16)				
**S**	**FIA**	**2**	1	1	N (16)	0	33.3	11.1	11.1
					N (14)				
					N (20)				
					N (17)				
					L (22)				
					L (18)				
					H (19)				
**ORF3a**	**ALS**	**3**	2	1	N (16)	33.3	NA^b^	NA	NA
					L (6)				
					H (14)				
**ORF3a**	**LLY**	**6**	2	4	N (16)	11.1	NA	NA	NA
					H (17)				
					H (4)				
					H (6)				
**ORF6**	**HLV**	**5**	3	2	L (19)	NA	NA	NA	NA

a Reported frequencies were calculated as follows: None, N (tested but no responses were detected in SARS-CoV-2-positive individuals); Low, L (>0%, <33% of responses detected in SARS-CoV-2-positive individuals); High, H (>33% of responses detected in SARS-CoV-2-positive individuals).

b NA, not applicable.

### *In Vitro* Priming of Human Antigen-Specific CD8^+^ T Cells

Thawed (peripheral blood mononuclear cells) PBMCs were resuspended at 10^7^ cells/ml in 24-well tissue culture plates (5 × 10^6^ cells/well) or in a 75-cm^2^ tissue culture flask (7 × 10^7^ cells/flask) containing AIM medium (Life Technologies, Monza, Italy) supplemented with FLT3L (50 ng/ml; Miltenyi Biotec, Bologna, Italy) to mobilize resident DCs, as previously described (23, 24). After 24 h (day 1), the peptides were added to the cultures, and DC maturation was induced with TNFα (1,000 U/ml, Miltenyi Biotec), IL-1β (10 ng/ml, Miltenyi Biotec), IL-7 (0.5 ng/ml, R&D Systems), and prostaglandin E2 (1 μM, Calbiochem, Milan, Italy). On day 2, (Fetal Bovine Serum) FBS (Euroclone) was added at a final v/v ratio of 10%. Medium was then replaced at days 4 and 7 with fresh RPMI 1640 (Euroclone, Milan, Italy) enriched with 10% FBS, non-essential amino acids (Euroclone) and sodium pyruvate (Sigma-Aldrich, Milan, Italy). Antigen-specific CD8^+^ T cells were characterized on day 10.

### ELISpot Assay

Human IFN*γ* ELISpot PLUS (HRP) kits with precoated plates were obtained from Mabtech (Nacka Strand, Sweden). PBMCs (1 × 10^5^ upon priming or 2.5 × 10^5^ for *ex vivo* stimulation) were seeded in duplicate and stimulated with peptide pools or matrixes. Cells incubated with medium alone were used as negative control, whereas those stimulated with an anti-CD3 monoclonal antibody (Mabtech) represented the positive control. Plates were incubated for 24 h, processed according to the manufacturer’s instruction, and read by an automated reading system (AELVIS, Hannover, Germany). The number of specific IFN*γ*-secreting T cells, expressed as spot-forming units (SFU) per 10^6^ cells, was calculated by subtracting the negative control values. Responses were considered after background subtraction, when more than 50 SFU/million cells were present. This threshold was taken after protocol optimization (25) and maintained throughout all assays. Results were excluded if the positive control was negative.

### Flow Cytometry

*Ex vivo* immunophenotype was performed on thawed PBMCs upon exclusion of dead cells with LIVE/DEAD Fixable Aqua (Life Technologies) (26) and the following directly conjugated monoclonal antibodies were used: anti-CD4 eFluor450, HLA-DR-APC, and anti-CD45RA PerCP-Cy5.5 (Life Technologies); anti-CD8 APC-Cy7 and anti-CCR7 PE-Cy7 (BD); anti CD27 FITC anti-PD1 PE (Miltenyi Biotec). Naive T cells were defined as CCR7^+^CD427^+^CD45RA^+^.

Intracellular cytokine staining was performed as previously described (27) with minor modifications. Briefly, 2 × 10^6^ PBMC_S_ were incubated with media alone or with the pool of 37 peptides for 6 h. Anti-CD107 Vioblue (Miltenyi Biotec) was added at the time of stimulation, whereas brefeldin and monensin were added after 1 h of stimulation. After incubation for 5 h at 37°C, cells were stained with LIVE/DEAD Fixable Aqua and anti-CD8 APC-Cy7. After fixation and permeabilization (Cytofix/Cyoperm, BD, Milan, Italy), cells were incubated with anti-IFN*γ* FITC (Life Technologies) and anti-TNF PE (BD) antibodies. Results obtained from cells incubated with media alone (NT) were subtracted from those obtained from cells incubated with the peptide pool (background subtraction). Boolean gates were performed on IFN*γ*, TNF, and CD107. Cytokine responses were background subtracted individually before further analysis. All acquisitions were performed on a FACS CANTO II (BD) upon compensation conducted with antibody capture beads (BD) (28). Flow cytometry data was analyzed using FlowJo (version 10.1, Tree Star Inc.).

### Protein Sequences and Alignment

Complete protein sequences for the envelope protein (E), membrane glycoprotein (M), nucleocapsid phosphoprotein (N), RNA dependent RNA polymerase (RdRp), spike protein (S), ORF3a, and ORF6 of SARS-CoV-2 and, when applicable, the HCoVs NL63, 229E, OC43, and HKU1, were downloaded from NCBI (accession numbers reported in **Supplementary Table S3**). MUSCLE algorithm was used to align multiple sequences.

### Statistical Analysis

Univariate statistical analyses were performed using non-parametric tests in Prism software version 9 (GraphPad Software Inc.). Statistical tests performed are described in figure legends. Significance was assigned at p <0.05.

## Results

### *In Vitro* Priming of SARS-CoV-2-Specific Naive CD8^+^ T Cells in Healthy Donors

**Figure 1:**
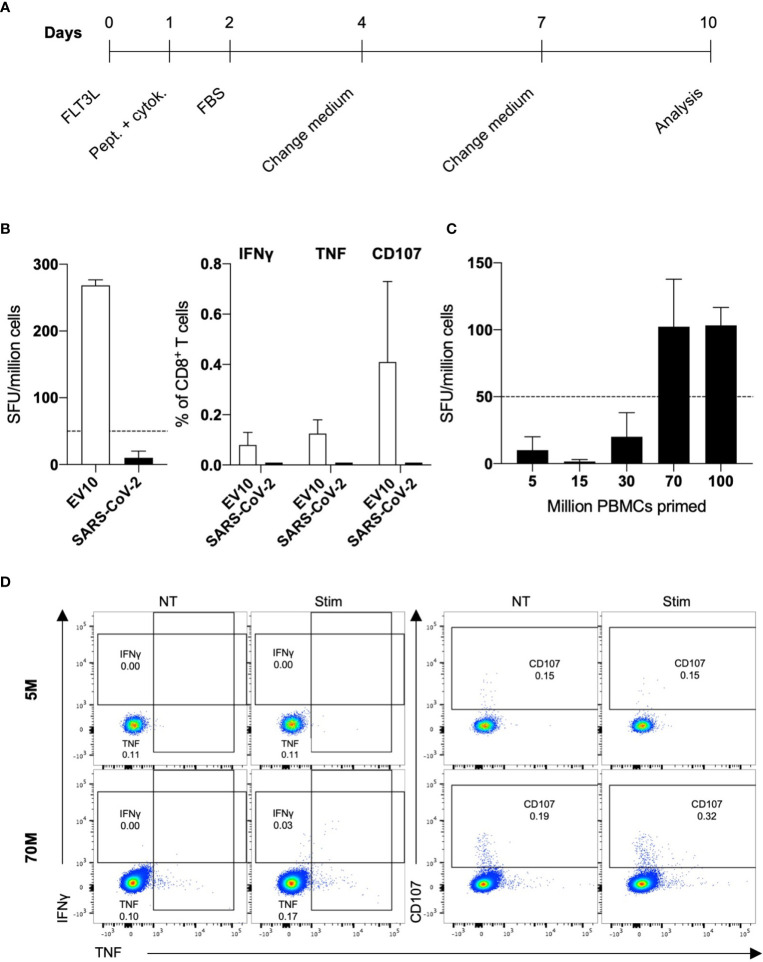
*In vitro* priming of SARS-CoV-2-specific naive CD8^+^ T cells in healthy donors. **(A)** Scheme of the *in vitro* priming protocol. **(B)** PBMCs (5 × 10^6^) were primed with EV10 or a pool of 37 SARS-CoV-2-derived peptides. After ten days, the frequency of peptide-specific primed naive CD8^+^ T cells was measured by IFN*γ* ELISpot (left), and the expression of IFN*γ*, TNF, and CD107 (right) was assessed by ICS. Data are shown, after background (NT) subtraction, as the mean + S.E.M. of two donors. **(C, D)** PBMCs (5–100 × 10^6^) were primed with a pool of 37 SARS-CoV-2-derived peptides. After ten days, the frequency of epitope-specific primed naive CD8^+^ T cells was measured by IFN*γ* ELISpot, and the expression of IFN*γ*, TNF, and CD107 was assessed by ICS. Data are shown, after background (NT) subtraction, as the mean + S.E.M. of three donors **(C)**. One representative dot plot for stimulation of 5 and 70 × 10^6^ PBMCs is shown **(D)**.

To assess the induction of primary SARS-CoV-2-specific CD8^+^ responses in unexposed healthy individuals, we exploited an *in vitro* system initially developed to prime naive T cells specific for the HLA-A2-restricted melanoma epitope EV10 in melanoma-naive donors (**Figure 1A**). This priming approach mirrors what happens *in vivo* in both mice and humans (10, 29).

In the present study, PBMCs from HLA-A2 positive blood donors were stimulated with a pool of 37 predicted and/or described HLA-A2-restricted peptide epitopes derived from different SARS-CoV-2 proteins (**Supplementary Table S1**) (6, 30–32). In parallel experiments, melanoma and SARS-CoV-2 peptides were used to prime naive CD8^+^ T cells. CD8-mediated responses were evaluated 10 days after stimulation by IFN*γ*-ELISpot and the expression of IFN*γ*, TNF, and CD107 was measured by intracellular cytokine staining (ICS). As shown in **Figure 1B**, EV10- but not SARS-CoV-2-specific CD8^+^ T cells were expanded. This *in vitro* priming protocol was developed to work with as few as 2.5 million PBMCs, exploiting the peculiar high frequency of naive CD8^+^ T cells specific for the EV10 epitope (33). Since the frequency of SARS-CoV-2-specific naive T cells is lower (15), ranging from one out 5 × 10^5^ to less than one out 10^7^, we escalated up to 100 million the number of PBMCs to be primed, without changing the conditions of readout assays. Starting from 70 million PBMCs, SARS-CoV-2-specific primary T-cell responses could be induced in unexposed donors (**Figures 1C, D** **)**.

### Reduced Antigenic Repertoire of SARS-CoV-2 Specific Primary CD8^+^ T Cell Responses in Older Adults

**Figure 2:**
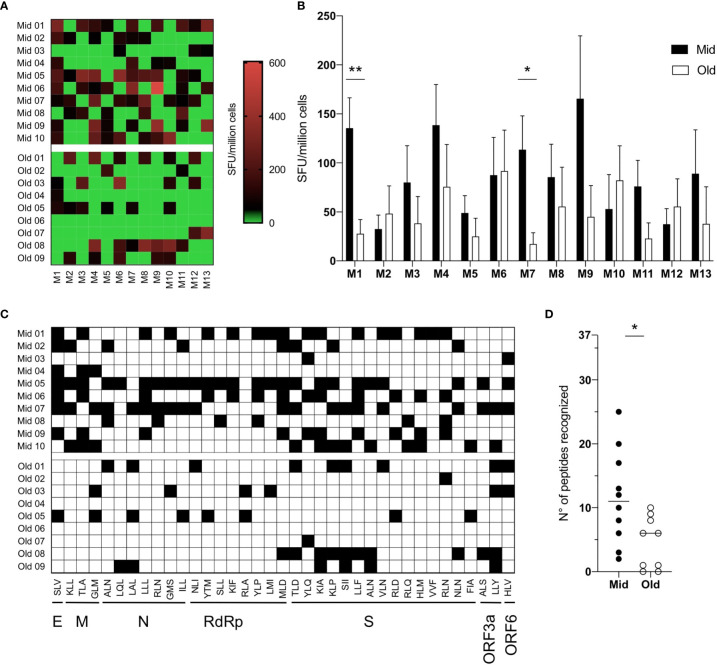
Reduced antigenic repertoire of SARS-CoV-2 specific primary CD8^+^ T-cell responses in older adults. **(A–D)** PBMCs (7 × 10^7^) were primed *in vitro* with a pool of 37 SARS-CoV-2-derived peptides. After ten days, the frequency of epitope-specific primed naive CD8^+^ T cells was measured upon restimulation with 12 different matrixes composed of six peptides each or with the YLQ peptide (matrix 13) by IFN*γ* ELISpot. Responses above the threshold of 50 SFU/million cells were considered positive. Individual **(A)** and median values + S.E.M. **(B)** of responses to single matrixes are shown. Since each peptide is contained in two different matrixes, when responses to both matrixes were above 50 SFU/million cells, the response toward that single peptide was counted as positive and marked in black **(C)**. The total number of recognized peptides by each donor was calculated and shown **(D)**; the lines represent the median values. Statistical significance was determined by Mann–Whitney test, *p < 0.05 and **p < 0.01.

We then exploited this *in vitro* priming system to test samples from HLA-A2 positive, SARS-CoV-2 unexposed healthy donors belonging to two age groups (**Supplementary Table S2**): middle-aged adults (Mid, median age 30-years-old, range 19–49, n = 10) and older adults (Old, median age 67–years-old, range 65–69 years, n = 9). To characterize the magnitude and breadth of the *in vitro*-induced responses, PBMCs from Mid and Old subjects were stimulated with the pool of the 37 peptides to prime SARS-CoV-2-specific naive CD8^+^ T cells. The cultures were tested 10 days after stimulation against a bidimensional peptide matrix system (**Supplementary Table S1**) by IFN*γ*-ELISpot. Data were first analyzed for the intensity of matrix-specific responses in the two age groups. As shown in **Figure 2A**, IFN*γ* release upon stimulation with single matrixes was generally higher in middle-aged adults. In particular, statistically significant differences were observed against M1 and M7 matrixes (**Figure 2B**). However, responses against some matrixes (*i.e.* M6 and M12) were similar between the age groups in terms of both magnitude and frequency (**Figures 2A, B** **)**. To assess the presence of cross-reactive responses due to memory T cells specific for common cold coronaviruses (HCoV), we stimulated PBMCs directly *ex vivo* with the same matrixes or with a pool of HLA-A2-presented epitopes derived from CMV, EBV, and HSV-1, as a control of recall responses. IFN*γ* secretion was measured 24* h* after stimulation. No positive responses toward the SARS-CoV-2 epitope matrixes were observed, while donors reacted to the pool of CMV, EBV, and HSV-1 peptides (**Supplementary Figure S1**).

We then used bidimensional matrixes to deconvolve the recognition of individual peptide epitopes by primed naive CD8^+^ T cells. This analysis showed that all peptides were recognized by at least one donor (**Figure 2C** and **Table 1**). The SLV E-derived peptide, the MLD RdRp-derived peptide, and the KIA S-derived peptide were the most frequently recognized (7–8 donors out of 19). Among these, responses against the SLV and MLD peptides were mostly detected in middle aged adults, which overall recognized a statistically significant higher number of peptides compared to older adults (**Figure 2D**). Of note, naive CD8^+^ T cell frequencies were statistically significantly lower in older adults and showed a positive correlation with the number of recognized peptides (although not statistically significant, **Supplementary Figure S2**). Other phenotypic baseline measurements, including activation levels assessed by HLA-DR and PD1 expression, were comparable among the age groups (**Supplementary Figure S2A**). Thus, although the readout of our experimental setting could also potentially capture alterations at the level of antigen presenting cells, these results suggest that the narrower responses observed in older subjects may be due to holes in the naive repertoire.

The percent of homology with the four HCoVs (NL63, 229E, OC43 and HKU1) was then computed. The majority of epitopes shared a similarity less than 50% (**Table 1**), while identical sequences were observed in some RdRp-derived peptides (RLA and YLP). However, we did not find any positive correlation between the percentage of homology and the numbers of donors responding to the peptides (**Supplementary Figure S3**), suggesting that cross-reactive immunity does not impact the CD8-mediated peptide-specific responses identified. Altogether, these data suggest that primary CD8^+^ T-cell responses to SARS-CoV-2 are quantitatively altered and narrower in older people.

### Altered Functions of Primed SARS-CoV-2-Specific CD8^+^ T Cells in Older Subjects

**Figure 3:**
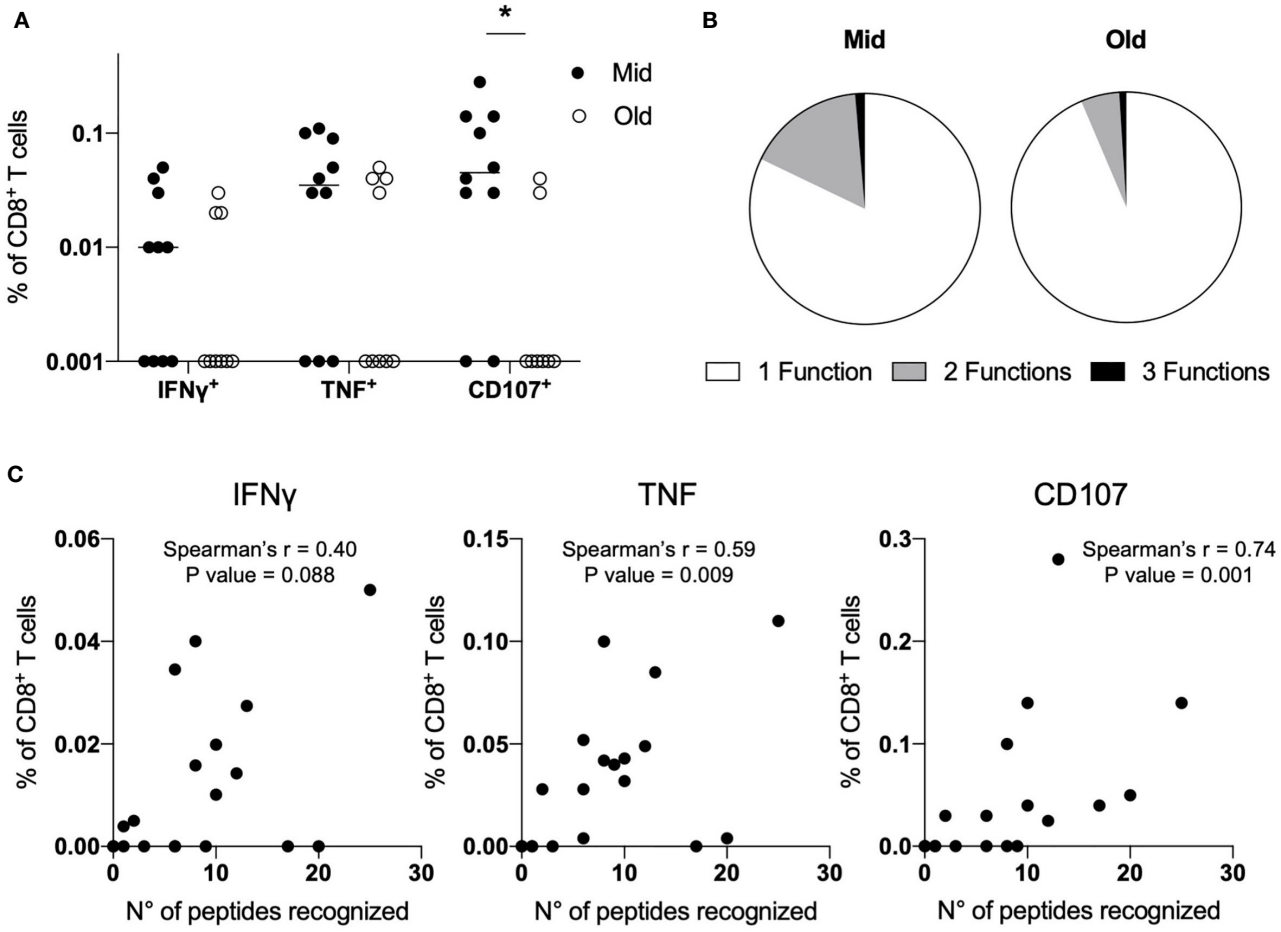
Altered functions of primed SARS-CoV-2-specific CD8^+^ T cells in older subjects. **(A–C)** PBMCs (7 × 10^7^) were primed in *vitro* with a pool of 37 SARS-CoV-2-derived peptides. After ten days, the frequency of epitope-specific primed naive CD8^+^ T cells was measured; upon restimulation with the same peptide pool, the expression of IFN*γ*, TNF, and CD107 was assessed by ICS. Data are shown as percentage of CD8^+^ T cells after background (NT) subtraction, and lines represent median values **(A)**. Polyfunctional capacity was determined and shown as a mean for Mid and Old donors **(B)**. The number of recognized peptides determined by IFNγ ELISpot was correlated with the production of IFNγ or TNF and the expression of CD107 determined by ICS **(C)**. Statistical significance was determined by Mann–Whitney test **(A)** and Spearman’s rank correlation **(C)**, *p < 0.05.

Finally, we sought to determine whether the age-associated alterations that we observed in naive CD8^+^ T cells primed with SARS-CoV-2 peptides could be appreciated evaluating the expression of IFN*γ*, TNF, and CD107 by ICS after stimulation with the pool of the 37 peptides. IFN*γ* production was generally very low, with a trend towards higher responses in middle-aged adults (**Figure 3A** and **Supplementary Figure S4**). The same pattern was observed for TNF production, although it reached higher levels compared to IFN*γ* in both age groups. Notably, the marker of degranulation CD107 was expressed at statistically significant higher levels in Mid compared to Old donors (**Figure 3A** and **Supplementary Figure S4**). Consistently with data from convalescent individuals (14, 32, 34, 35), we observed a high proportion of SARS-CoV-2-specific monofunctional CD8^+^ T cells, especially in older subjects (**Figure 3B**). Of note, the frequency of TNF^+^ and CD107^+^ CD8^+^ T cells directly correlated with the number of recognized peptides (**Figure 3C**). The same was observed with respect to IFN*γ* production, although results did not reach statistical significance. Together, these data confirm that middle-aged individuals have the capacity to mount superior *de novo* T-cell responses against SARS-CoV-2 compared to older subjects.

## Discussion

Immune aging is characterized by the presence of few and dysfunctional naive T cells (10, 36), and their loss is associated with severe COVID-19 clinical manifestations (2). This suggests that elderly individuals mount poor *de novo* responses toward SARS-CoV-2. To address this issue, we developed an *in vitro* approach to study the priming of SARS-CoV-2-specific CD8^+^ T-cell responses from naive cells in healthy unexposed individuals of different age groups, without the influence of the infection or comorbidities in infected patients. To our knowledge, SARS-CoV-2-specific CD8^+^ T-cell responses have been rarely detected in healthy donors, as studied so far, stimulating low cell numbers with the aim of identifying cross-reactive memory responses (15, 17, 22). In our study, we used 8–10 amino acid long peptides with a rather low homology with HCoVs. Of note, the majority of SARS-CoV-2-specific responses were against peptides with a minimal degree of homology. Furthermore, in a 24 h stimulation assay which should activate cross-reactive HCoVs-specific memory CD8^+^ T cells (37), we did not observe any detectable recall response. Although the expansion of low-differentiated memory cells in our *in vitro* priming system cannot be totally ruled out, we would exclude its major contribution, consistently with recent reports of a limited impact of HCoV exposure in shaping CD8^+^ T-cell responses (4, 20, 38, 39). Nonetheless, the issue of cross-reactive immunity deserves further investigations since memory CD4^+^ T cells specific for common cold coronaviruses and cross-reacting to SARS-CoV-2 have been frequently reported in uninfected individuals (20, 38, 40–43).

Among the 37 selected peptides, some were recognized by >30% of donors. However, the immunodominance pattern observed upon *in vitro* priming does not mirror that found in infected patients (**Table 1**). For example, the YLQ epitope, although immunodominant in infected patients, is not frequently recognized in unexposed controls. Similarly, the RLQ epitope, also found as immunodominant in convalescent patients, is recognized very rarely upon priming. Notably, in infected individuals, the response towards the YLQ epitope is more diverse than that toward the RLQ epitope (22). Thus, the inability to prime RLQ-specific naive T cells may derive by the low frequency of their clones. Considering the narrowing T-cell repertoire in old people, it is not surprising that we could only observe once the priming of YLQ-specific primary responses, and never RLQ-specific ones, in subjects >65-years-old. Conversely, and in agreement with Shomuradova’s studies, we observed the induction of primary responses toward the SII and NLN epitopes, which are subdominant in infected individuals (22). Distinct immunodominant patterns between infected and healthy individuals after T-cell expansion using overlapping 15mers covering the N, NSP17, and NSP13 proteins were also reported by Le Bert et al. (40). These data suggest that immune responses towards certain epitopes are preferentially selected during the infection. Considering that several factors, including viral load and infection duration, determine the magnitude and the breadth of CD8^+^ T-cell responses (30), the precise causes behind this shift of the repertoire require more in-depth analysis.

The T-cell response against SARS-CoV-2 seems to be mainly dominated by CD4^+^ T lymphocytes (44, 45). Nevertheless, cytotoxic T cells have been shown to be important for protection (4, 5, 7). Low disease severity is strongly associated with higher number of SARS-CoV-2-specific CD8^+^ T cells and of naïve CD8^+^ T cells (2). Although few studies investigated age-specific patterns of antiviral immunity, it has been shown that elderly infected patients harbor low frequencies of virus-specific CD8^+^ T cells (4), and age correlates with few IFN*γ*-producing T cells (2, 8). Our data indicate a poor priming capacity of SARS-CoV-2 specific CD8^+^ T-cell responses in healthy older subjects. Furthermore, we demonstrate that primary cellular responses are directed toward a low number of peptide epitopes in older subjects, consistently with the holes in the T-cell repertoire occurring with aging (9). Although we did not use overlapping peptides spanning the whole sequence of the seven antigens targeted in our study, we could appreciate that some peptides, such as the SLV E-derived peptide, 2 M-derived peptides (KLL and TLA) and the MLD RdRp-derived peptide were recognized more by younger subjects. Some S-derived peptides were nonetheless recognized at similar frequencies in the two age groups (*e.g.* SII and ALN). This suggests that older individuals may still mount *de novo* responses against this antigen, even if narrower and potentially suboptimal, not arguing against immunization campaigns targeting the elderly. Further studies are however necessary to investigate the magnitude, duration, and protection levels of long-term responses induced by the natural infection or by the vaccine in the most-at-risk populations. In the meantime, it is crucial to pay special attention to older subjects, even when vaccinated.

## Data Availability Statement

The original contributions presented in the study are included in the article/**Supplementary Material**. Further inquiries can be directed to the corresponding author.

## Ethics Statement

The studies involving human participants were reviewed and approved by Regional health authority (AUSL). Written informed consent for participation was not required for this study in accordance with the national legislation and the institutional requirements.

## Author Contributions

EG, RG, and FN designed the study. RG and FN coordinated the project. EG, DP, BD, and MC performed the experiments. MC performed the bioinformatics analyses. SP, VAl, and EM, synthetized the peptides. SP, VAl, PM, AC, and VAp provided reagents and feedback on the methods and manuscript. FN analyzed the data. RG and FN wrote the manuscript. All authors contributed to the article and approved the submitted version.

## Funding

This study was supported by the CRASH CAGE - One-Health Target project, funded by the Ludwig Maximillians University, Center for International Health through a grant from the Exceed Program of German Academic Exchange Services (DAAD).

## Conflict of Interest

The authors declare that the research was conducted in the absence of any commercial or financial relationships that could be construed as a potential conflict of interest.
