# Supplementary material for: Impaired Priming of SARS-CoV-2-Specific Naive CD8+ T Cells in Older Subjects
Source: Front Immunol. 2021 Jul 13;12:693054. doi: 10.3389/fimmu.2021.693054 (PMC8315546; doi:10.3389/fimmu.2021.693054)
Supplement: Supplementary file 1 [file DataSheet_1.docx]

Supplementary Material


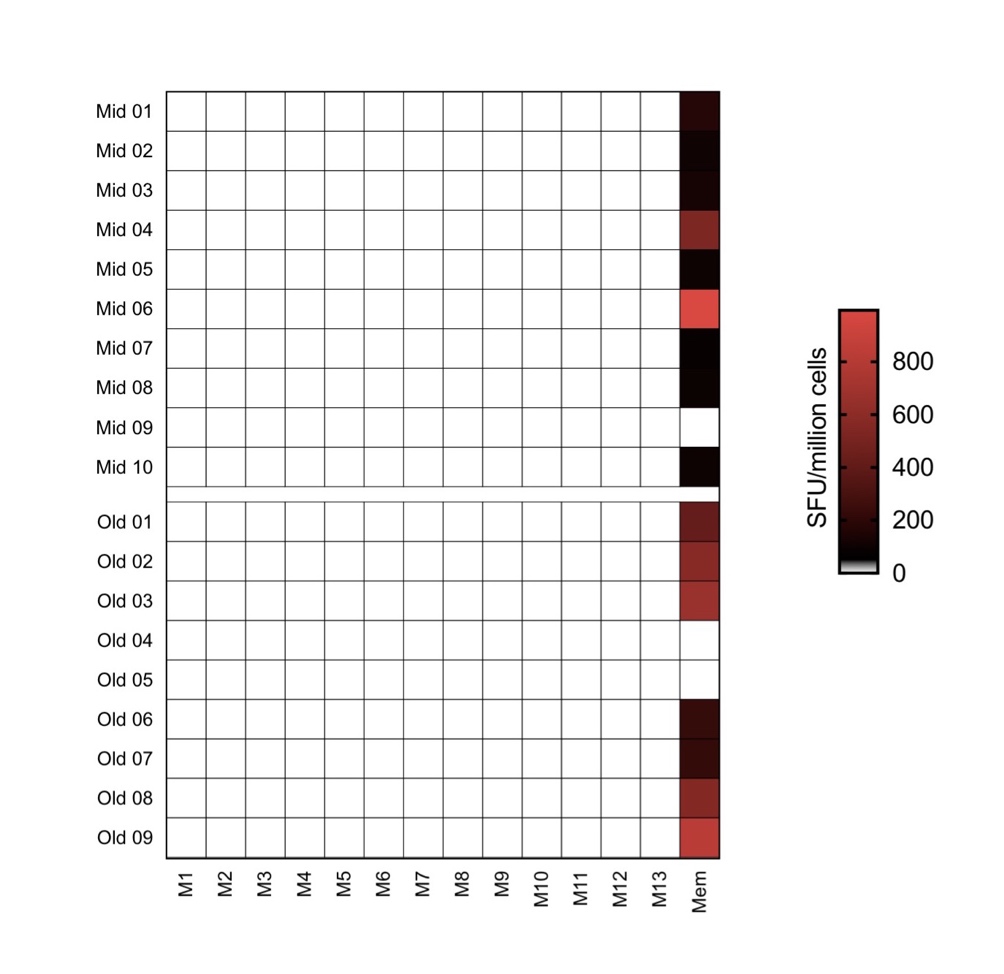


**Supplementary Figure 1.**

**Absence of memory CD8^+^ T cells cross-reacting to SARS-CoV-2-derived peptides**

PBMCs (2.5 x 10^5^/well) were stimulated for 24 hours with 12 different matrixes composed by 6 peptides each, with the YLQ peptide (M13) or with a pool of peptides from CMV, EBV and HSV-1 (Mem). IFNγ release was measured by ELISpot. Responses above the threshold of 50 SFU/million cells were considered positive. The magnitude of matrix responses are indicated in red scale.

**Supplementary Figure 2.**

**Differentiation and activation phenotypes of study subjects**

(**A**) PBMCs of study subjects stratified by age were analyzed for the expression of differentiation markers (CD45RA, CD27 and CCR7) to identify naïve T cells and of activation markers (PD1 and HLA-DR). (**B**) The correlation between the number of peptides recognized determined by IFNγ ELISpot and the percentage of naïve CD8^+^ T cells is shown. Statistical significance was determined by Mann Whitney test (A) and Spearman’s rank correlation (B), *p<0.05.

**
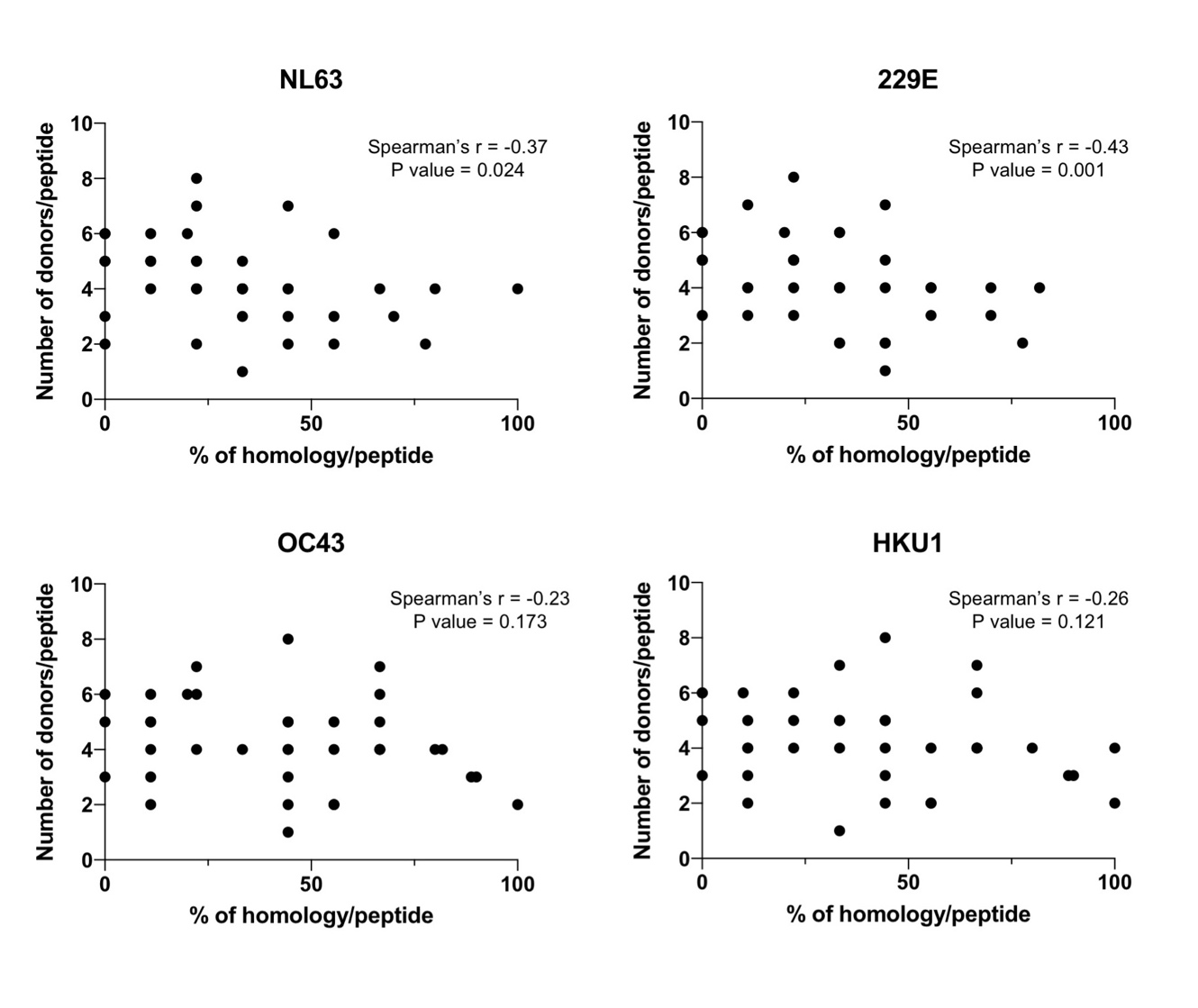
**

**Supplementary Figure 3.**

**Correlation between peptide homology and number of responders to the priming**

The percentage of homology between SARS-CoV-2-derived peptides and the 4 HCoVs NL63, 229E, OC43 and HKU1 was calculated and correlated with the number of donors showing positive responses toward each peptide upon priming. Statistical significance was determined by the Spearman’s rank correlation.


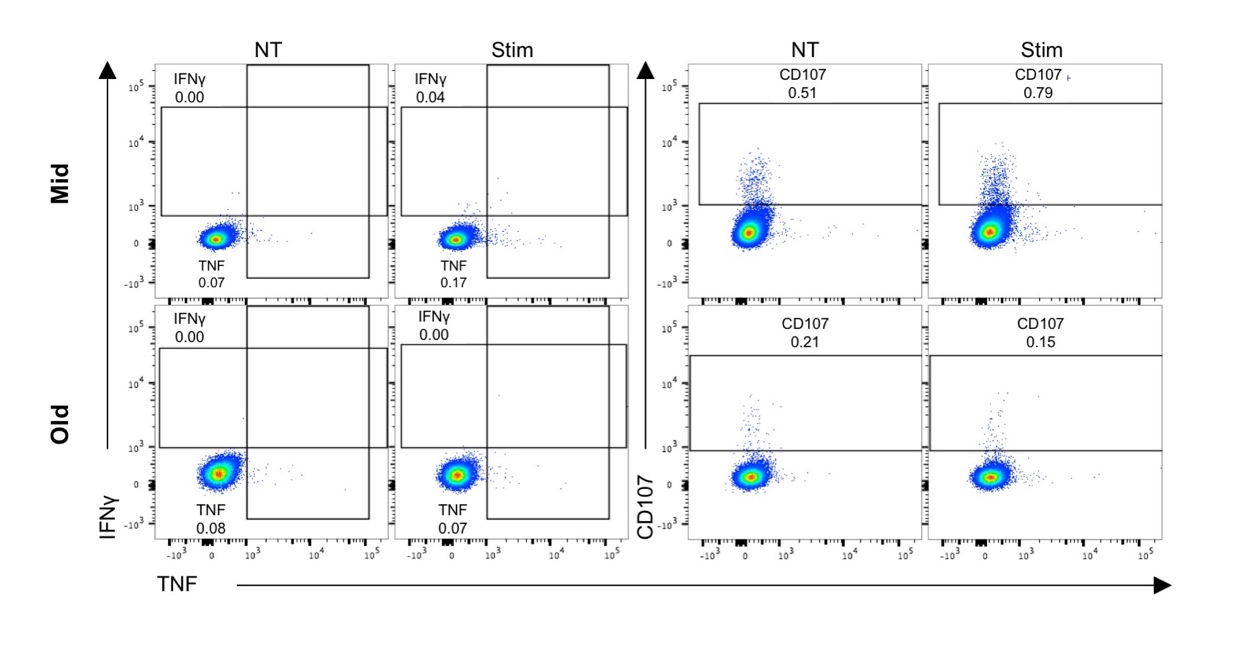


**Supplementary Figure 4.**

**Age impacts on the functions of primed SARS-CoV-2-specific CD8^+^ T cells**

PBMCs (7 x 10^7^) were primed *in vitro* with a pool of 37 SARS-CoV-2-derived peptides. After ten days, the frequency of epitope-specific primed naïve CD8^+^ T cells was measured, upon restimulation with the same peptide pool, assessing by ICS the expression of IFNγ, TNF and CD107. Representative dot plots for Mid and Old are shown.

**Supplementary Table 1.**

**HLA-A2-restricted, SARS-CoV-2-derived peptides used in the study**

| **Protein** | **Code** | **Sequence** | **Start-end** | | **Length** | **Matrix** | **Predicted in** |
| --- | --- | --- | --- | --- | --- | --- | --- |
| E | **SLV** | SLVKPSFYV | 50- | 58 | 9 | 1, 7 | (6, 22, 23) |
| M | **KLL** | KLLEQWNLV | 15- | 23 | 9 | 1, 8 | (6, 22, 23) |
| M | **TLA** | TLACFVLAAV | 61- | 70 | 10 | 1, 9 | (6, 21) |
| M | **GLM** | GLMWLSYFI | 89- | 97 | 9 | 1, 10 | (6) |
| N | **ALN** | ALNTPKDHI | 138- | 146 | 9 | 2, 8 | (6) |
| N | **LQL** | LQLPQGTTL | 159- | 167 | 9 | 2, 9 | (6) |
| N | **LAL** | LALLLLDRL | 219- | 227 | 9 | 2, 10 | (6) |
| N | **LLL** | LLLDRLNQL | 222- | 230 | 9 | 1, 11 | (6, 21-23) |
| N | **RLN** | RLNQLESKM | 226- | 234 | 9 | 2, 11 | (6) |
| N | **GMS** | GMSRIGMEV | 316- | 324 | 9 | 1, 12 | (6, 21) |
| N | **ILL** | ILLNKHIDA | 351- | 359 | 9 | 2, 7 | (6, 21) |
| RdRp | **NLI** | NLIDSYFVV | 4456- | 4464 | 9 | 2, 12 | (6, 22) |
| RdRp | **YTM** | YTMADLVYAL | 4514- | 4523 | 10 | 3, 7 | (6, 22) |
| RdRp | **SLL** | SLLMPILTL | 4631- | 4639 | 9 | 3, 8 | (6, 22) |
| RdRp | **KIF** | KIFVDGVPFV | 4724- | 4733 | 10 | 3, 9 | (6, 22) |
| RdRp | **RLA** | RLANECAQV | 5046- | 5054 | 9 | 3, 10 | (6, 22) |
| RdRp | **YLP** | YLPYPDPSRIL | 5220- | 5230 | 11 | 3, 11 | (6, 22) |
| RdRp | **LMI** | LMIERFVSL | 5246- | 5254 | 9 | 3, 12 | (6, 22) |
| RdRp | **MLD** | MLDMYSVML | 5291- | 5299 | 9 | 4, 7 | (6, 22) |
| S | **TLD** | TLDSKTQSL | 109- | 117 | 9 | 6, 12 | (23) |
| S | **YLQ** | YLQPRTFLL | 269- | 277 | 9 | 13 | (6, 21-23) |
| S | **KIA** | KIADYNYKL | 417- | 425 | 9 | 4, 8 | (6, 22) |
| S | **KLP** | KLPDDFTGCV | 424- | 433 | 10 | 6, 7 | (6) |
| S | **SII** | SIIAYTMSL | 691- | 699 | 9 | 4, 9 | (6, 22) |
| S | **LLF** | LLFNKVTLA | 821- | 829 | 9 | 4, 10 | (6, 22) |
| S | **ALN** | ALNTLVKQL | 958- | 966 | 9 | 6, 8 | (6) |
| S | **VLN** | VLNDILSRL | 976- | 984 | 9 | 4, 11 | (6, 21-23) |
| S | **RLD** | RLDKVEAEV | 983- | 991 | 9 | 4, 12 | (6, 22) |
| S | **RLQ** | RLQSLQTYV | 1000- | 1008 | 9 | 5, 7 | (6, 22) |
| S | **HLM** | HLMSFPQSA | 1048- | 1056 | 9 | 5, 8 | (6, 22) |
| S | **VVF** | VVFLHVTYV | 1060- | 1068 | 9 | 5,11 | (6) |
| S | **RLN** | RLNEVAKNL | 1185- | 1193 | 9 | 5, 10 | (6, 21) |
| S | **NLN** | NLNESLIDL | 1192- | 1200 | 9 | 5, 12 | (6, 21) |
| S | **FIA** | FIAGLIAIV | 1220- | 1228 | 9 | 5,9 | (6, 21, 22) |
| ORF3a | **ALS** | ALSKGVHFV | 72- | 80 | 9 | 6, 10 | (6, 23) |
| ORF3a | **LLY** | LLYDANYFL | 139- | 147 | 9 | 6, 9 | (6, 23) |
| ORF6 | **HLV** | HLVDFQVTI | 3- | 11 | 9 | 6, 11 | (6, 23) |

**Supplementary Table 2.**

**Study subjects**

| **Subject** | **Sex** | **Age** |
| --- | --- | --- |
| Mid 01 | F | 19 |
| Mid 02 | M | 21 |
| Mid 03 | M | 23 |
| Mid 04 | M | 23 |
| Mid 05 | M | 26 |
| Mid 06 | M | 33 |
| Mid 07 | M | 35 |
| Mid 08 | M | 36 |
| Mid 09 | F | 39 |
| Mid 10 | M | 49 |
| Old 01 | M | 65 |
| Old 02 | F | 65 |
| Old 03 | F | 65 |
| Old 04 | M | 65 |
| Old 05 | M | 66 |
| Old 06 | F | 66 |
| Old 07 | M | 69 |
| Old 08 | M | 69 |
| Old 09 | M | 69 |

**Supplementary Table 3.**

**Protein identities used**

| **Protein** | **Virus** | | | | |
| --- | --- | --- | --- | --- | --- |
|  | **SARS-CoV-2** | **NL63** | **229E** | **OC43** | **HKU1** |
| E | YP_009724392.1 | YP_003769.1 | [NP_073554.1](https://www.ncbi.nlm.nih.gov/protein/12175751) | YP_009555243.1 | [YP_173240.1](https://www.ncbi.nlm.nih.gov/protein/56807328) |
| M | YP_009724393.1 | [YP_003770.1](https://www.ncbi.nlm.nih.gov/protein/45655912) | [NP_073555.1](https://www.ncbi.nlm.nih.gov/protein/12175752) | YP_009555244.1 | [YP_173241.1](https://www.ncbi.nlm.nih.gov/protein/56807329) |
| N | YP_009724397.2 | YP_003771.1 | NP_073556.1 | YP_009555245.1 | YP_173242.1 |
| RdRp | YP_009724389.1 | AIW52828.1 | QNT54753.1 | YP_009555260.1 | [YP_459941.1](https://www.ncbi.nlm.nih.gov/protein/85719076) |
| S | YP_009724390.1 | [YP_003767.1](https://www.ncbi.nlm.nih.gov/protein/45655909) | NP_073551.1 | YP_009555241.1 | [YP_173238.1](https://www.ncbi.nlm.nih.gov/protein/56807326) |
| ORF3a | YP_009724391.1 | [YP_003768.1](https://www.ncbi.nlm.nih.gov/protein/45655910) | - | - | - |
| ORF6 | YP_009724394.1 | - | - | - | - |
